# 3D Modeling of Building Indoor Spaces and Closed Doors from Imagery and Point Clouds

**DOI:** 10.3390/s150203491

**Published:** 2015-02-03

**Authors:** Lucía Díaz-Vilariño, Kourosh Khoshelham, Joaquín Martínez-Sánchez, Pedro Arias

**Affiliations:** 1 Applied Geotechnologies Research Group, University of Vigo. Rúa Maxwell s/n, Campus Lagoas-Marcosende, Vigo 36310, Spain; E-Mails: joaquin.martinez@uvigo.es (J.M.-S.); parias@uvigo.es (P.A.); 2 Faculty of Geo-Information Science and Earth Observation, University of Twente, P.O. Box 217, Enschede 7514 AE, The Netherlands; E-Mail: k.khoshelham@utwente.nl; 3 Department of Infrastructure Engineering, University of Melbourne, Melbourne 3010, Australia

**Keywords:** 3D modeling, feature extraction, openings, imagery, LiDAR data, BIM

## Abstract

3D models of indoor environments are increasingly gaining importance due to the wide range of applications to which they can be subjected: from redesign and visualization to monitoring and simulation. These models usually exist only for newly constructed buildings; therefore, the development of automatic approaches for reconstructing 3D indoors from imagery and/or point clouds can make the process easier, faster and cheaper. Among the constructive elements defining a building interior, doors are very common elements and their detection can be very useful either for knowing the environment structure, to perform an efficient navigation or to plan appropriate evacuation routes. The fact that doors are topologically connected to walls by being coplanar, together with the unavoidable presence of clutter and occlusions indoors, increases the inherent complexity of the automation of the recognition process. In this work, we present a pipeline of techniques used for the reconstruction and interpretation of building interiors based on point clouds and images. The methodology analyses the visibility problem of indoor environments and goes in depth with door candidate detection. The presented approach is tested in real data sets showing its potential with a high door detection rate and applicability for robust and efficient envelope reconstruction.

## 1. Introduction

In recent years, 3D building reconstruction has become a research subject of interest due to the increasing demand for up-to-date building models, which are requested as an input source for a variety of purposes.

The application area for which buildings are reconstructed determines both geometric detail and semantic content of the models. On the one hand, realistic 3D city models are gaining importance because three-dimensional virtual reality is much more efficient than 2D data for applications such as urban planning, global energy analysis—e.g., heat island effect or wind tunnels—evacuation routes or city navigation for tourism purposes. In these large-scale applications, the representation of the exterior of buildings in the form of 2.5D elevation models or block models with textures that provide a realistic visualization of façades may be enough. On the other hand, there is an increasing need for highly detailed 3D building models with information about their interior structure. The representation of building interiors, where we spend a great deal of our time, is important for a variety of applications from architectural planning, to lighting and energy analysis, crowd management or even crime scene investigation. Specifically, 3D modeling of building interiors including details such as doors and windows is very important for applications like indoor navigation, emergency route planning and crisis management in large indoor environments

Representation of the modeled objects can be carried out according five different levels of detail [1]. The coarsest level of detail is the LoD-0 in which buildings are defined as 2.5D representations. The LoD-1 represents building as prismatic block models with vertical walls and horizontal roofs while in the LoD-2 the shape of roofs is represented as well as walls and balconies. LoD-3 is the most detailed level for exterior description in which holes such as windows and doors are included and finally, LoD-4 includes the representation of building interiors.

As manual creation of a building model is a time-consuming process that requires expert knowledge [2], reconstruction tools based on the interpretation of measured data, such as LiDAR data and images, are frequently used to automatically extract building geometry.

There is a wide range of approaches for automatically modeling buildings with the coarsest levels of detail, from LoD-0 to LoD-2. Regarding the reconstruction of large-scale cities with a LoD-2, a review of a number of approaches for city modeling and building reconstruction, in order to detail the state of the art of the techniques and their grounds, is available [3]. Recently, the reconstruction of buildings and their immediate surroundings from Terrestrial Laser Scanner data was presented in [4], resulting in LoD-2 models for solar analysis purposes. Nevertheless, recent efforts aim to enrich building models in order to obtain more complex and realistic representations (LoD-3 and LoD-4). While in the first case (LoD-3), façades have to be modelled in a more complex way by including windows, doors and other protrusions such as balconies, corbels, *etc.*; in the second case, building interiors have to be reconstructed.

The challenge in indoor modeling is automated reconstruction of LoD-4 models. This requires methods that can accurately reconstruct structural elements (walls, floors and ceilings) in presence of noise, occlusion and clutter, but can also robustly recognize and model important details, particularly doors. Existing approaches rely mostly on point clouds as the main source of data for geometric reconstruction of interiors, and recognize doors as openings in the point cloud. This requires that the doors are open during data acquisition, and eventually some closed doors will be missed during the reconstruction. The contribution of this paper is to propose an approach based on combined use of point cloud data, acquired by a laser scanner, and image data, captured by an off-the-shelf digital camera. We present a data-driven method for geometric reconstruction of structural elements using the point cloud, and a model-driven method for the recognition of closed doors in image data based on the generalized Hough transform.

### 1.1. Related Work

According to [5], reconstruction methodologies can be classified into two major categories: data-driven (non-parametric or bottom-up) approaches and model-driven (parametric or top-down) approaches. While data-driven approaches directly extract features such as points or edges from data, model-driven approaches use previous knowledge to search for the most appropriate model from a specific library [6] and fitting it to the data [7].

Despite the presence of significant clutter and occlusions, which frequently occur in building interiors, some approaches have dealt successfully with the reconstruction of indoor spaces [8,9] and their structural elements (walls, floors and ceilings) [10–13] from imagery and/or point cloud data. A good review about building reconstruction procedures according to this classification was presented in [14]. Therefore, this section is specifically focused on openings reconstruction.

#### 1.1.1. Data-Driven Approaches

As mentioned above, data-driven techniques consider data as the unique input source for modeling buildings regardless of form. Therefore, they strongly depend on data quality. Since geometrical features are directly extracted and modeled from the measurements without previous knowledge, these approaches are also relatively sensitive to clutter and occlusions [15].

When a point cloud is used for reconstruction and assuming homogeneous point cloud density, a large number of these approaches bases the location of openings on searching holes or low data density regions on the wall plane. On the one hand, windows and doors can be assumed as holes because they are usually not coplanar with the wall in which they are contained, especially in case of façades. Thus, after carrying out a segmentation process, both windows and doors can be extracted from the boundary points of the holes on the façade segment [16–18]. On the other hand, a laser beam usually penetrates window glasses, so that no laser points are reflected, causing areas with low raw laser information. [9,19] consider this fact for classifying point cloud voxels with a ray-tracing algorithm into three categories: *opening* (windows or doorways), *occupied* and *occluded.* Analyzing data density and classifying low-density areas as openings limit the scope to low-density windows and doorways.

Other approaches are based on the use of color information. An illustration of such methods is the recognition and labeling of windows from thermal colored 3D point clouds [20]. As the material and the thermal conductivity is different in walls and in windows, a temperature map is used for detecting windows and labelling them as *closed*, *open* or *damaged*.

#### 1.1.2. Model-Driven Approaches

In contrast to data-driven methods, model-driven approaches integrate previous knowledge about the appearance and arrangement of object structures. They are more robust in the presence of partial occlusion, since they incorporate some form of knowledge about the shape of the object [15]. Model-driven approaches have been widely used in computer vision for object recognition and classification in images, and most of them can be classified into two categories: generative and discriminative strategies. Generative based methods organize the meaningful parts of the input data for stochastically obtaining different models. After a predicted model is generated and comparted with real data, the parameters of the predicted model are modified in order to archive the highest degree of similarity. Discriminative based methods use statistical analysis and machine learning techniques to learn template characteristics from training data. The authors in [21] propose a categorization of façade openings, windows, doors and balconies, without supervision using Bag-of-Words models. Another interesting approach for window detection using Implicit Shape Models is proposed by [22]. Preliminary experiments on door detection using the Generalized Hough Transform (GHT) to estimate the parameters of rectangular shape models have been presented [23].

### 1.2. Proposed Approach

**Figure 1.**
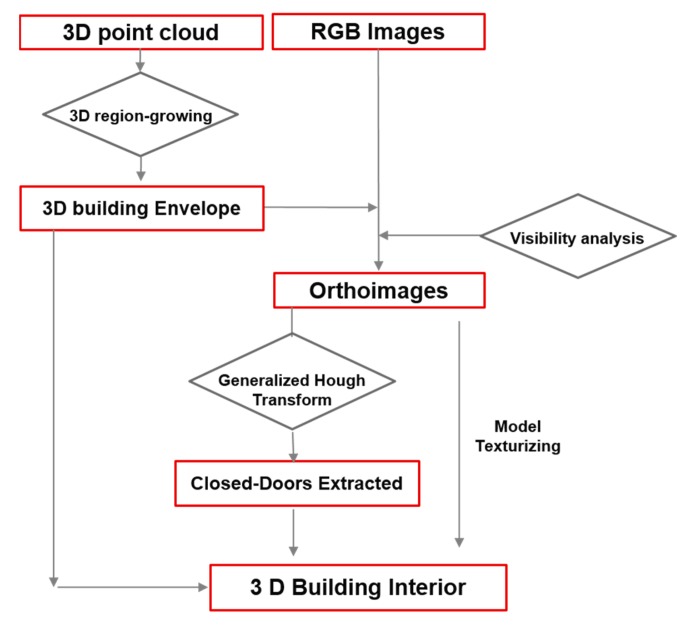
Schema of the proposed methodology for building interior reconstruction.

In this work, we propose an automatic approach for the reconstruction of LoD-4 models, consistent with a data-driven method for geometric reconstruction of structural elements using the point cloud, and a model-driven method for the recognition of closed doors in image data based on the generalized Hough transform (Figure 1). The approach starts with the segmentation of the point cloud based on a curvature analysis followed by a 3D region-growing algorithm. Data is provided by a Terrestrial Lasers Scanner with a high-resolution digital camera mounted on top. In comparison with other sensors such as RGB-D cameras (*i.e.*, Microsoft Kinect), which are being explored in depth for mapping indoors [24], and Terrestrial Laser Scanners (TLS), which have the capability to acquire point clouds with higher geometric accuracy and a wider range of working distances [25]. Then, a visual labelling is carried out for reconstructing the building envelope. The geometry of walls is used for the orthoimages generation, which are the basis for the closed-door detection approach. The final step consists in projecting the doors extracted from the orthoimages onto the 3D building envelope in order to obtain the complete 3D building interior models with geometrical and identity information.

This paper is organized as follows. Section 2 introduces data acquisition devices and data sets while Section 3 explains the methodology developed for the building interior reconstruction. Section 4 is focused on presenting the results and the discussion obtained from the application of the methodology to four cases of study, and finally, Section 5 deals with the conclusions of the work.

## 2. Instruments and Data

**Figure 2.**
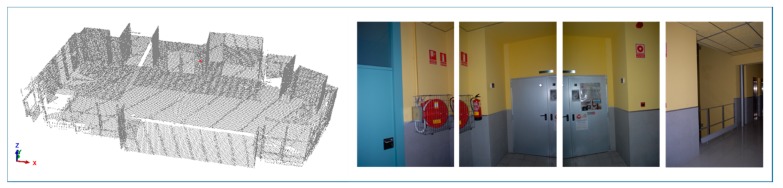
An example of a complete point cloud of an indoor scene and four images partially covering the horizontal field of view.

Data sets consist of point clouds and images obtained from a single hybrid acquisition system formed by a Terrestrial Laser Scanner (TLS), model Riegl LMS Z-390i (RIEGL Laser Measurement Systems GmbH, Horn, Austria), and a high-resolution digital camera (Nikon D200, Nikon Corporation, Tokyo, Japan) firmly mounted on top (Figure 2).

The technical characteristics of the laser device used in this work are summarized in Table 1. The laser scanner presents a field of view of 360° horizontally and 80° vertically, which implies missing information from the immediate ceiling and floor on top and under it. This fact, together with the complex geometry of the different indoor scenes and the presence of objects between the scanner and the indoor envelope that provoke occlusions, makes data acquisition from different positions necessary. The number of scanner positions and their location is determined by the user according to the shape complexity of the indoor scene, maximizing the area of interest visible from each of them.

Many laser scanner devices have a photographic camera incorporated in their configuration. However, the TLS used in this work integrates a calibrated photographic camera on top. Although last-generation cameras have lower levels of noise, the adopted Nikon D200 is quiet enough for the purpose of the present work. On the one hand, the Internal Calibration Parameters are calculated following the photogrammetric calibration process of self-calibration bundle adjustment based on flat check pattern images [26,27]. Table 2 shows the intrinsic and the main internal calibration parameters of the photographic device.

On the other hand, the External Orientation Parameters of the camera are automatically obtained through a point matching process using reflective targets. Therefore, the geometric relation between each laser point and the camera is always known. A series of coordinate systems, which define the geometric dependence between the sensors and a higher order reference system, are the basis of all coordinate transformations [28]. The origin of the local coordinate system is settled in the origin of one of the laser scanner positions. The acquisition procedure is analogous for every building case and consists of two phases: firstly, a low-resolution scanning (0.2°) of the entire scene (360° horizontally) provides data from the visible area from the scan position point-of-view, and secondly, a higher density scanning is performed on the area of interest with an angular resolution of 0.08°. The position of the scanner and the number of Scan Positions depend on the complexity of the interior scene.

Generally, the *Z*-axis of the laser device is vertically aligned during the acquisition procedure. However, the reduced dimensions of building interiors make necessary to tilt the mount for completing the 360° of the vertical field of view of the scanner from the same position. In the first and second cases of study, the device was tilted with −30° and +40°, respectively.

Once all data sets were registered in the same coordinate system through a point matching process using reflective targets, they were submitted to a pre-processing step, the results of which are shown in Table 3.

**Figure 3.**
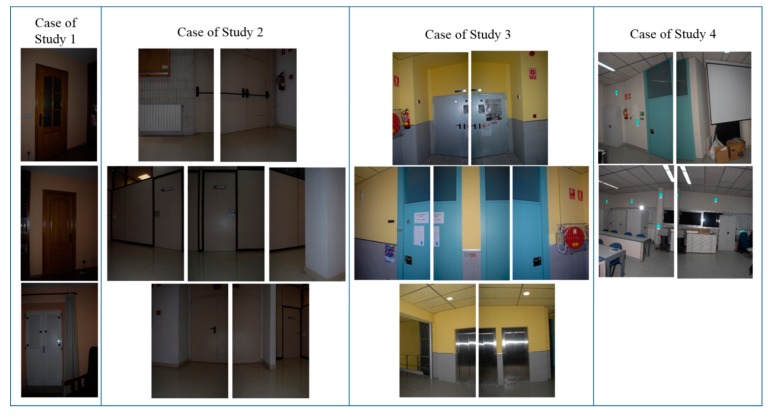
The first three cases of study present different kinds of closed doors. The last building interior (case of study 4) contains just one door but also pieces of furniture with closed doors.

Regarding image acquisition, the field of view of the device used allows the capture of the complete scene with 10 images, with a 10% overlap between consecutive pictures. All the cases of study were selected according to the criteria of maximizing the number and variability of closed doors for testing the robustness of the approach. Figure 3 shows the different kind of doors available in the four building interiors. While in the first, second and third cases of study there are three types of distinguishable closed doors, in the fourth case, there is just one. However, the last scene is still challenging because it contains some pieces of furniture with similar shape and size to doors.

## 3. Methodology

This section includes the steps of the proposed methodology for the automatic reconstruction of 3D indoor scenes. The initial subsection (Section 3.1) includes the preceding steps required for the closed door extraction, which are both reconstruction of the indoor envelope and true-orthoimage generation. Afterwards, Section 3.2 deals with the extraction of closed doors based on the Generalized Hough Transform.

### 3.1. Interior Envelope Generation and Orthorectification of Images

**Figure 4.**
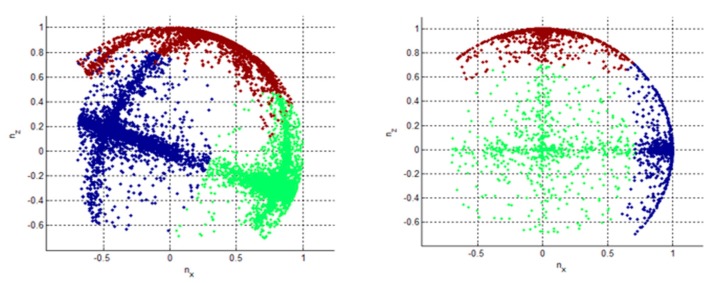
Normal vectors are clustered in three groups according to the *x* (blue); *y* (green) and *z* axes (red); before (**left**) and after (**right**) the rotation procedure.

In order to simplify the subsequent generation of a number of orthoimages corresponding to walls, the procedure starts with a point cloud rotation, in a way that floor and ceiling are parallel to the *x*-*y* plane and walls are parallel to either *x*-axis or *y*-axis, if possible. The necessary rotation angles can be estimated from the distribution of the normal vectors of the points. The normals are clustered using the *k-means* algorithm into three groups. The three cluster centers form a 3D rotation matrix from the *x*-*y* aligned coordinate system to the original point cloud coordinate system. The inverse rotation is used to align the point cloud with the Project Coordinate System axes (Figure 4). $R={\left[\begin{array}{ccc} {\bar{\text{n}}}_{\text{x}}^{1} & {\bar{\text{n}}}_{\text{x}}^{2} & {\bar{\text{n}}}_{\text{x}}^{3}\\ {\bar{\text{n}}}_{\text{y}}^{1} & {\bar{\text{n}}}_{\text{y}}^{2} & {\bar{\text{n}}}_{\text{y}}^{3}\\ {\bar{\text{n}}}_{\text{z}}^{1} & {\bar{\text{n}}}_{\text{z}}^{2} & {\bar{\text{n}}}_{\text{z}}^{3} \end{array}\right]}^{\text{T}}$ , where n̄^1^, n̄^2^, n̄^3^ are the three cluster centers.

The procedure continues with a segmentation of the point cloud based on a seeded region-growing method [29], by which planar surfaces that form the indoor space are detected.

**Figure 5.**
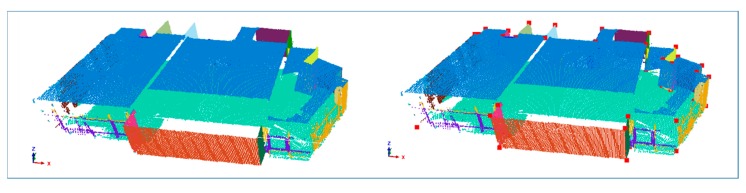
The segmented regions of an indoor scene are individually shown in different colors (**left**); Once regions are identified and intersected, boundary points defining each surface (red color) are obtained (**right**).

In order to minimize the effect of noise on the normal values, a smoothing is performed by averaging the normal vector of points in the same neighborhood (*k*) [30]. The point with the lowest curvature value is chosen as the region seed candidate for each iteration and the region is growing if an angular and a distance point-to-plane conditions are satisfied. Moreover, a minimum number of points in the region is set for filtering false positives (Figure 5, left). Afterwards, horizontal regions are automatically classified into “ceiling” and “floor” according to the Z component of their normal and centroid, while vertical regions are submitted to a visual inspection for their identification and labelling. Then horizontal and vertical planes are intersected in order to achieve the boundary points that define each surface (Figure 5, right).

**Figure 6.**
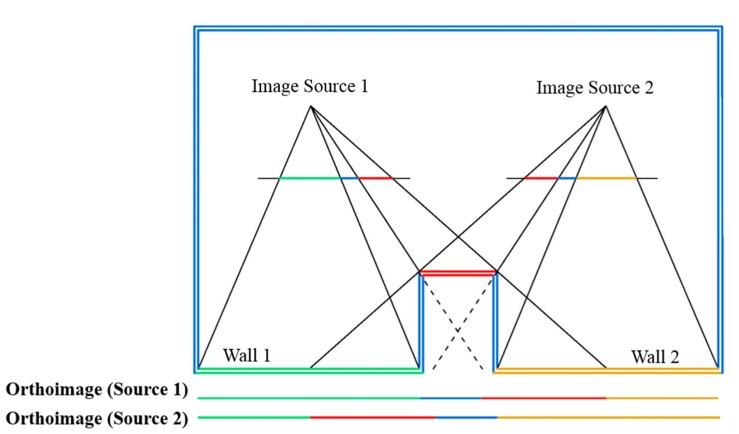
The visibility problem in concave angles.

Building indoors are generally composed of vertical walls with rectangular shapes. Therefore, the four 3D boundary points that define each wall can be used as the basis for a true-orthoimage generation. True-orthophoto generation has been extensively studied [31,32]. In true-orthophoto generation, the source images are projected over a surface model taking into account its visibility. Visibility problem is a major computer graphics topic and several approaches have been developed in order to solve it [33,34]. Compared to other 3D objects, walls are relatively simple structures. If two consecutive walls form an angle bigger than 180°, visibility condition is not fulfilled from any point of view of the interior scene (Figure 6).

**Figure 7.**
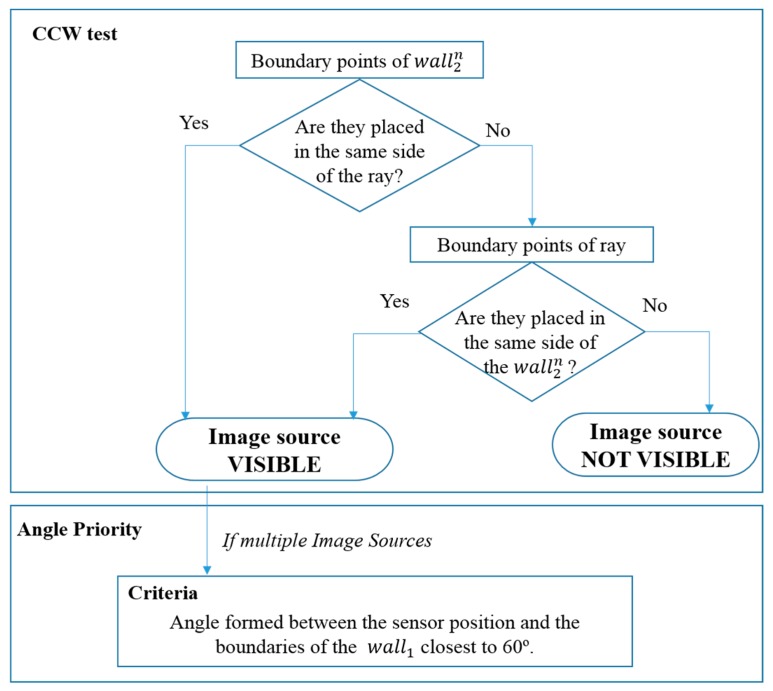
Schema of the visibility analysis.

Visibility analysis is carried out through a simple ray-tracing algorithm enough to check if walls forming building interiors are visible from the source images and to avoid a double mapping effect that occurs when a back-projection of the points is performed on a wrongly selected image-space (Figure 7).

**Figure 8.**
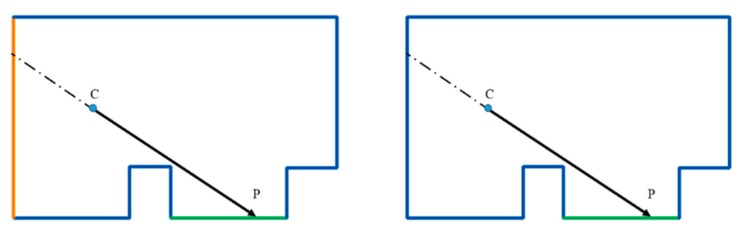
Schema of the CCW test.

For each pixel in an image (*i.e.*, *wall^1^*), a ray in the object space towards the projection center of the camera is defined. The remaining walls are considered as potential occlusion walls ( $wall_{n}^{2}$ ) and, as they are typically vertical elements, they are submitted to a 2D Counter Clock Wise test (CCW) for knowing on which side of the ray are placed [35]. If boundaries of the potential occlusion walls ( $wall_{n}^{2}$ ) are placed on the same side in relation to the ray, visibility is guaranteed (blue walls in Figure 8, left). If this condition is not satisfied (orange wall in Figure 8, left), the location of the edges of the ray is studied. According to this, if the edges of the ray are placed on the same side regarding the position of the wall, visibility is also ensured (Figure 8, right).

Thereupon, the best image-source is selected among the images, which are visible from the point-of-interest. In a simple case where only one image source is used to acquire color data, this step can be omitted. However, in cases of multiple image sources, the horizontal angle of view formed between the sensor position (C) and the boundaries of the wall is used for establishing a priority order. The optimal position for the sensor is forming an angle closest to 60°. In order to avoid the ghost-effect, the points on a wall share a common image-priority order unless the visibility criteria is not accomplished.

**Figure 9.**
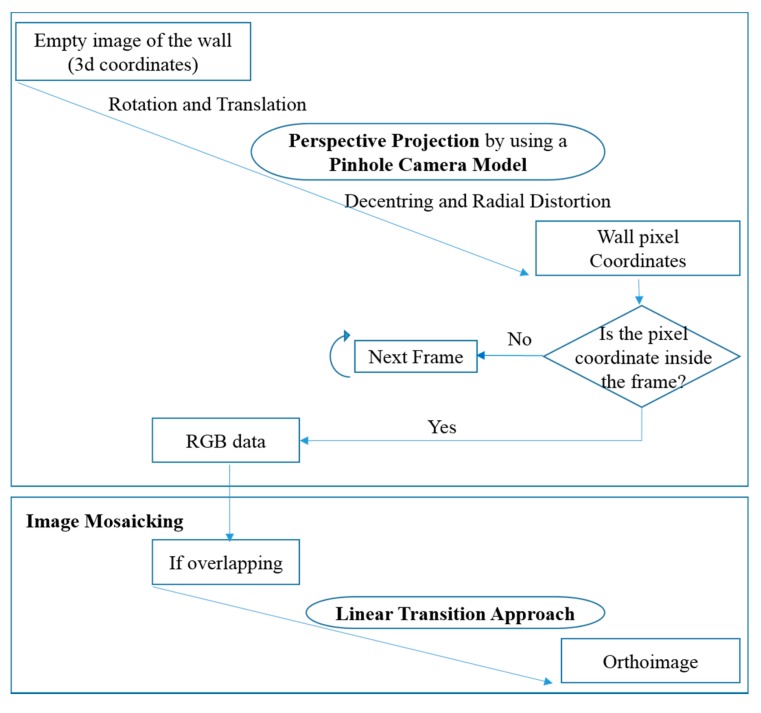
Schema of the procedure of orthoimages generation.

After defining the orthoimage plane and image resolution and selecting the most suitable image source according to a visibility criteria, RGB values are obtained through the perspective projection of the 3D points using a pinhole camera model [36]. Orthoimage generation is shown in Figure 9.

Lens distortion is calculated and fixed to make possible the correction of the difference between the actual camera projection and the camera model, which is introduced by the lens. The Interior Orientation Parameters are applied to the computation of the radial and decentering corrections, which are undone to obtain the distorted pixel coordinates. Finally, as image acquisition is done with a 10% overlapping between consecutive images, a Linear Transition Method is implemented in order to eliminate edge seams caused by a direct pixel average fusing [37]. This method can make the pixel in the overlapping area transitioned from the first image to the second image slowly, which provokes the smoothness of the transition part with no obvious edge seam.

### 3.2. Closed Door Extraction

The extraction of closed doors is carried out through an image-based algorithm applying the Generalized Hough Transform to the orthoimages generated in the previous step. The approach is invariant to scale changes and can handle reasonably small occlusions and noise.

**Figure 10.**
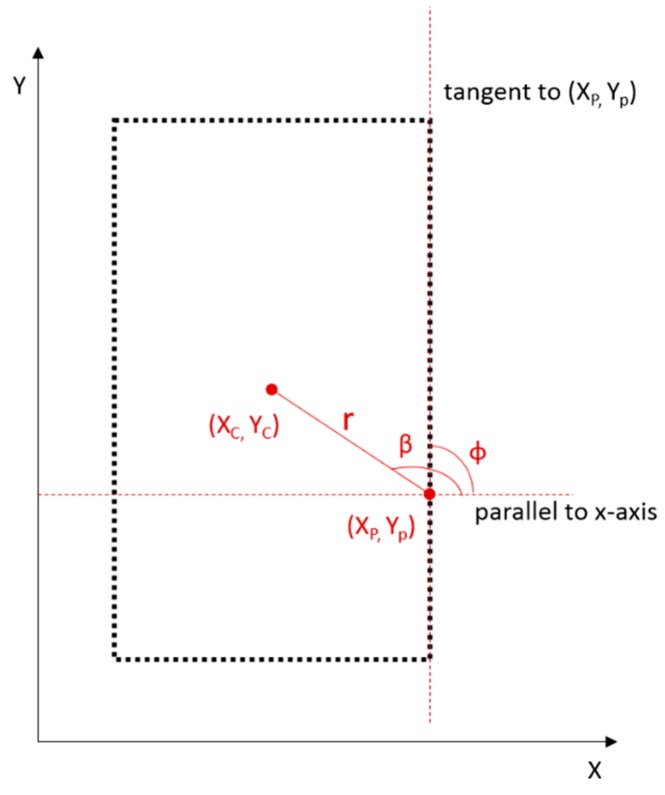
Parameters involved in the GHT for door-candidate detection.

The Generalized Hough Transform uses a model shape to find similar patterns in an image. We use a rectangle as our model and store it in a so-called R-table. The R-table is essentially a look-up table, in which every point on the rectangle is represented by its edge orientation φ and a vector defined by (r, β) to an arbitrary center point (Figure 10). The φ values are used as index to look up the vectors to the center point.

Through a voting process we find rectangular patterns of edge pixels in the image. Therefore, first step consists on converting true color orthoimages to grayscale images, where edges are found by using the *Canny* operator. As closed doors can be assumed as rectangles vertically oriented, resulting edges are submitted to an orientation filter through which only horizontally and vertically oriented edge pixels are retained for the subsequent computation.

The detection of rectangular candidates is carried out as follows:

1. Construct a 4 dimensional accumulator over (X_C_, Y_C_, S_x_, S_y_) where S_x_ and S_y_ are the scale parameters corresponding to width and height of a door.
2. For each edge pixel in the image with an orientation value φ, look up the matching entry in the R-table and retrieve all the vectors (r, β) corresponding to this entry.
3. Voting: for each pair of (r, β) and for different scale parameters:
  - a. Calculate the coordinates of the center point (X_C_, Y_C_).
  - The coordinates of each center point and the two scale parameters are used to cast a vote in the 4D accumulator. Local maxima in the accumulator represent the parameters of candidate rectangles in the image.

In order to enforce the detection of door candidates, some additional constraints are taken into account: minimum and maximum width and height, doors vertically oriented, as well as distance between neighbor candidates.

**Figure 11.**
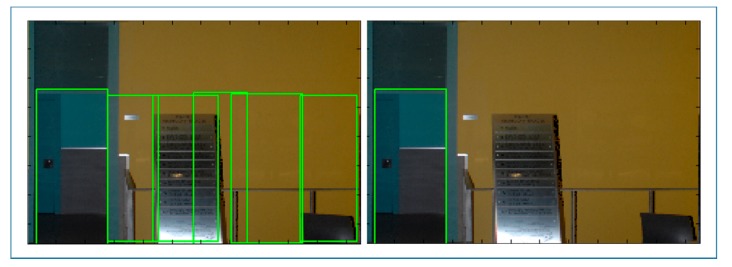
The results of the door extraction are shown before (**left**) and after (**right**) the selection.

Given that the number of doors in each wall is not known, a higher number of bins than expected are searched in each orthoimage resulting in an over-detection of door candidates (Figure 11, left). In this search, neighbors of most voted bins are suppressed within a certain threshold for avoiding redundancy.

Finally, door candidates are submitted to a local and global selection based on their voting rate. First, each orthoimage is considered individually and the bins with a voting rate inferior to a local percentile are deselected. Next, all orthoimages are considered together and the bins with a voting rate superior to the global percentile are selected. In this way, the resulting doors are the most voted candidates for each wall in the context of the whole indoor building (Figure 11, right).

**Figure 12.**
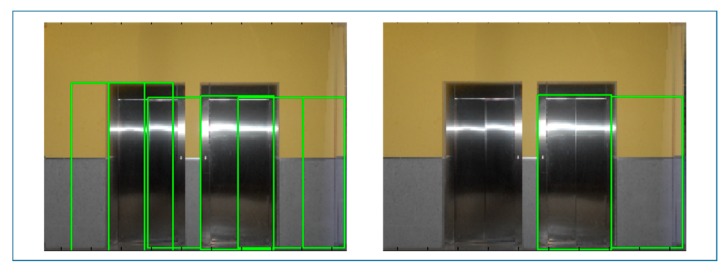
The results of the door extraction in a failure case.

Figure 12 shows a failure case where, after submitting door candidates (left) to the voting selection, the most voted candidates consist on a true and a false positive (right).

Final step consists on projecting the rectangles detected in the orthoimages on the 3D building envelope in order to get the 3D coordinates of the closed doors.

## 4. Results and Discussion

### 4.1. Interior Envelope Generation and Orthorectification of Images

Segmentation of building interior surfaces is carried out from the registered and filtered point clouds. The main results of this process are shown in Table 4. The angle threshold of region growing step is higher in the two first building interiors (80°) than in the third one and fourth one (75°). This is because the 1st and 2nd case studies are formed by walls, ceiling and floor orthogonally connected as opposed to 3rd and 4th cases which present some non-orthogonally connected walls. In addition, the 3rd case of study presents a lower value of local connectivity because it is formed by some non-connected walls with similar Y component.

Despite the grouping carried out by the region-growing step, there are residual regions that come from over-segmentation. A high over-segmentation rate makes the region recognition more difficult [30]. These residual regions are manually inspected and corrected. The estimated exploration time is minimal compared to a manual modeling of the whole scene.

The final regions are manually recognized and intersected in order to create the boundary representation model of the scenes. These models are submitted to a geometric quality analysis in terms of areas and distances. According to this, the ceiling surface of each building case is selected for area analysis and the height and width of the first wall for linear analysis. The comparison is made by taking the respective point cloud as ground truth. Results are shown in Table 5. Evaluating the results, the median error is −0.69% and −0.28% in area and distance measurements, respectively.

Walls are defined by four 3D boundary points ordered in counterclockwise direction, starting from the lower-left point from an indoor point of view. Geometry of walls is the basis for orthoimages generation, which is carried out considering a resolution of two centimeters because it allows a successive processing in reasonable time without affecting the quality of results.

**Figure 13.**
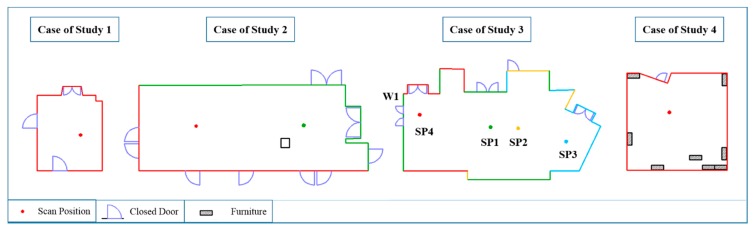
Scan Position selection for each wall according to the angle prioritizing and pixel-visibility test.

Visibility analysis is accomplished in those building cases where acquisition was performed from two or more scan positions. Visibility check is carried out for every pixel and scan positions are prioritized for each wall according to the 60° angle criteria. The most suitable scan position according to the priority criteria is selected for providing RGB data to all the pixels of each wall, as long as the visibility condition is ensured. Nevertheless, if the visibility condition is not satisfied for the most suitable scan position, the successive scan positions are used. The Scan Positions selected for each wall are showed in Figure 13.

**Figure 14.**
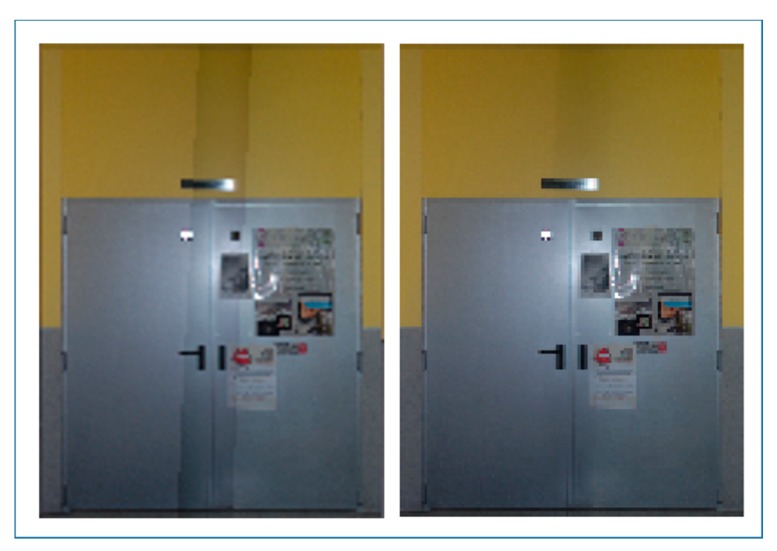
Left orthoimage presents edge seams as opposed to right orthoimage, where pixels are fused making the overlapping area transited from the first image to the second image slowly.

Finally, one orthoimage for each wall is generated according to the central perspective projection. Furthermore, in those cases in which two or more images are combined, edge seams are eliminated by applying the Linear Transition method for both horizontal and vertical overlapping. Figure 14 shows an orthoimage of case study 3 before (left) and after (right) edge seams elimination.

**Figure 15.**
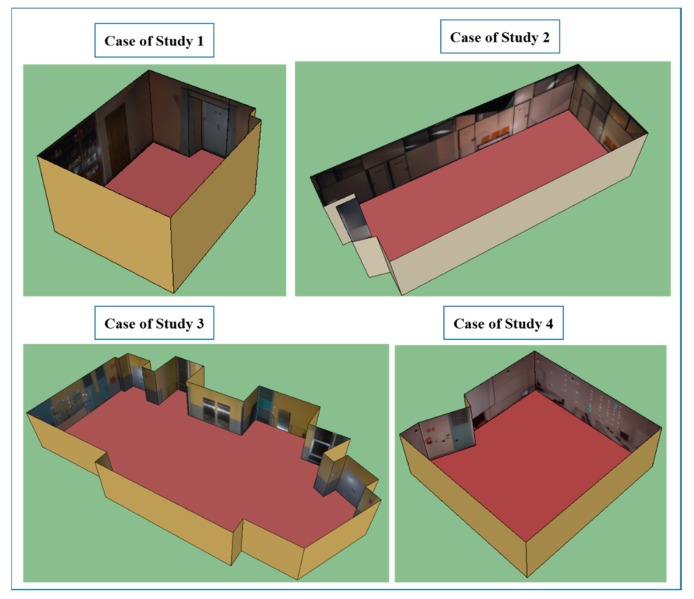
3-D building interiors are exported to Sketchup^©^ with the resulting orthoimages.

In Figure 15, orthoimages, which are the inputs of closed door extraction, are shown as textures the 3D envelope of each case study.

### 4.2. Closed Door Extraction

**Figure 16.**
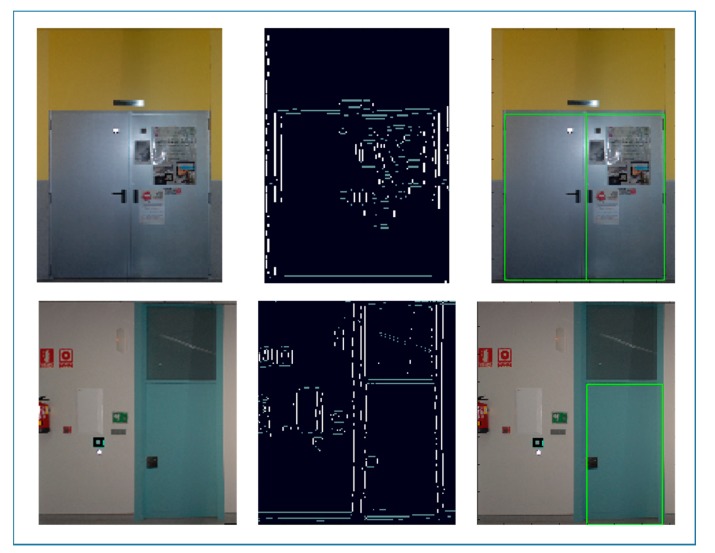
Two door examples from case study 3 (**up**) and case study 4 (**down**): the input orthoimages (**left**); the resulting edge images after horizontal and vertical edge pixel selection (**medium**) and the door candidates detected (**right**).

Finally, orthoimages are submitted to the door detection approach. All orthoimages of each building case are processed together, so that, the input parameters are common to all of them. As explained in Section 3.2, orthoimages are converted to grayscale images for finding edges with *Canny* operator. As doors are assumed as rectangles vertically oriented, the resulting edges are submitted to an orientation filter through which 90° and 180° oriented edge pixels are selected for computation. As it can be observed in Figure 16, the presence of additional elements such as posters causes clutter in the edge image. Furthermore, the lowest contrast between door and the bottom part of the wall incurs in missing door boundary edges. Both the presence of additional elements in the orthoimage as well as the lowest contrast between door and other objects may influence the detection process. However, as GHT detects shapes based on a maximum analysis, it is robust to partial occlusion and clutter caused by the presence of additional structures in the image [15].

Given that the number of doors in each wall is not known, 25 bins are searched in each orthoimage resulting in an over-detection of door candidates. Neighbors of most voted bins are suppressed within a 10 cm threshold for avoiding redundancy. Finally, door candidates are submitted to a local and global selection based on their voting rate as explained in Section 3.2. The results of each cases study are shown in Table 6.

In order to analyze the results, we design an evaluation scheme based on four commonly used properties: precision, recall and F1-score. Precision indicates the correctness of the door identification, so that, true positives are evaluated with regard to total, both true and false positives. Recall represents the ability to identify doors correctly, where true positives are compared with existent doors in each building test. F1-score combines recall and precision with equal weights [38].

The results of the methodology evaluation are shown in Table 7. Doors are correctly detected as true positives in the first and forth case study. However, in the second and third experiments, two doors are missing (false negatives). In reality, these false positives are detected by the GHT. However, in both case studies, the number of door candidates, understood as rectangles detected after performing the GHT, is very high due to the great color similarity between doors and walls (case study 2) and to the existence of furniture and curtain walls (case study 3). This fact causes that missing doors obtain a lower percentage of votes compared to false positives and they are excluded during the local and global selection. Finally, false positives are obtained in the third and fourth case studies. The main reason is the presence of curtain walls in third case and furniture with similar shape and size, such as cupboards, bookshelves, in the fourth case.

**Figure 17.**
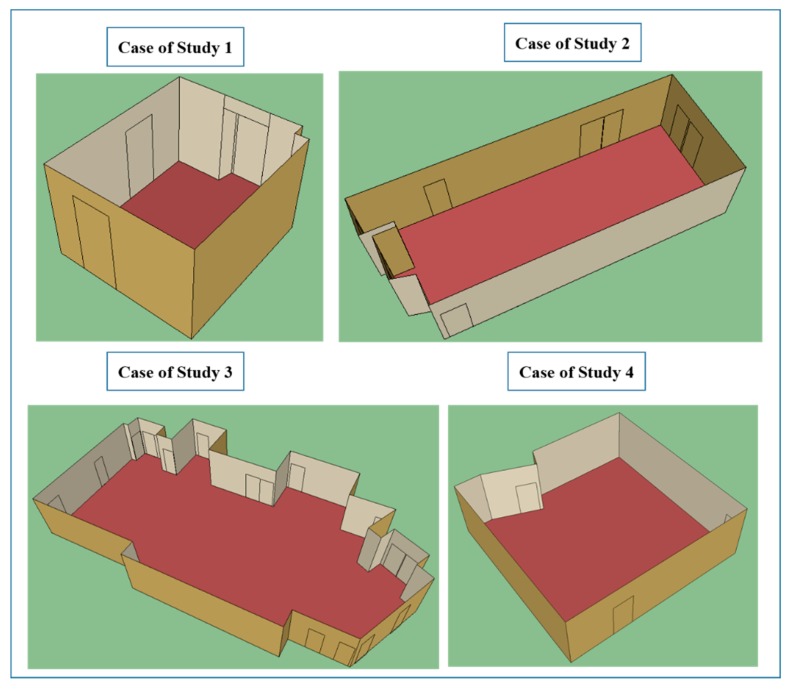
The resulting 3D building interiors visualized in Sketchup^©^.

Finally, the resulting doors are shown as three dimensional elements included in their correspondent building envelope (Figure 17).

## 5. Conclusions

The paper presents a pipeline of techniques for the reconstruction and interpretation of building interiors. While the point cloud is the basis for modeling the envelope of the scene, closed doors are extracted from the orthoimages.

From the results, the following main conclusions can be drawn:

- The proposed methodology optimizes the results of the 3D model by maximizing the information acquired without increasing acquisition time, given that both the point cloud and the corresponding images are captured at the same time.
- The building interior modeling process is robust enough for envelope reconstruction without submitting data to a manual cleaning and thus, minimizing the processing time.
- The visibility analysis based on a ray-tracing algorithm optimizes the generation of orthoimages selecting the most suitable image sources, avoiding wrong RGB projections and orthoimage areas without information. These high quality orthoimages are not only used for detecting doors but also to texture the models and create real-looking visualizations.
- The extraction of closed doors is carried out with a robust approach to clutter and occlusions. All the doors are initially detected as candidates by the GHT. Even though additional constraints lead to results that are not 100% accurate and complete, the method can largely reduce the amount of manual work in modeling doors.
- False negatives and false positives can be obtained especially if other elements with the same shape and size as doors are present in the building interior.

The point cloud could be used for excluding false positives from doors with similar size and shape. In case of curtain walls, the point cloud density could be analyzed because windows are typically low-density areas. In case of pieces of furniture, a point-to-plane distance should be obtained for knowing the position of the candidate with regard to the correspondent wall plane. If it is coplanar within a certain threshold, the candidate would be a true positive.

In summary, the combination of images and point clouds enables the reconstruction of building components, especially in the case of closed doors in which geometry is not enough to detect them.

## Figures and Tables

**Table 1.** Technical characteristics of the Riegl LMS Z-390i laser scanning device according to the manufacturer datasheet.

**Technical Characteristics**	
Measurement range	From 1 to 400 m
Accuracy (at 50 m)	6 mm
Repeatability (at 50 m)	4 mm
Vertical scanner range	0–80°
Horizontal scanner range	0–360°
Angular resolution	0.002°–0.2°
Measurement rate (points per second)	8000–11,000
Laser wavelength	Near infrared

**Table 2.** Technical specifications of the Nikon D200 camera.

**Technical Characteristics**	
Camera Model	Nikon D200
Lens Model	Nikon 20 mm
Pixel size	6.1E−6 × 6.1E−6 m
Pixels	3872 × 2592 pix
Focal length	3357.27 pix
Position Principal Point	1926.24 × 1322.28 pix

**Table 3.** Acquisition and pre-processing results.

	**Case of Study 1**	**Case of Study 2**	**Case of Study 3**	**Case of Study 4**
Total Scan Positions	1	4	4	1
Tilted Scan Positions	1 (−30°)	-	2 (+40°)	-
Average Error Registration	-	0.0031 m	0.0034 m	-
Original number of points	1,969,622	3,745,843	5,389,396	1,296,318
Octree	0.02 m	0.08 m	0.08 m	0.05 m
Points after Pre-Processing	187,361	82,736	152,045	86,628

**Table 4.** Segmentation results.

	**Case of Study 1**	**Case of Study 2**	**Case of Study 3**	**Case of Study 4**
Angle Threshold (*θ_th_*)	80°	80°	75°	75°
Local Connectivity (*d_th_*)	0.15 m	0.15 m	0.08 m	0.15 m
Number of Segmented Regions	14	12	34	8
Over-Segmented Residual Regions	4	2	6	0
Surfaces Submitted to Visual Identification	10	10	28	9

**Table 5.** The results of geometric quality analysis of building envelope reconstruction.

**Ceiling**	**Case Study 1**	**Case Study 2**	**Case Study 3**	**Case Study 4**
Area (automatically)	15.68 m^2^	57.12 m^2^	195.62 m^2^	99.60 m^2^
Area (ground truth)	15.69 m^2^	58.62 m^2^	194.15 m^2^	100.97 m^2^
Error	−0.01%	−2.56%	+0.01%	−1.36%

Wall 1				

Height (automatically)	2.50 m	2.93 m	3.06 m	3.26 m
Height (ground truth)	2.52 m	3.01m	3.04 m	3.23 m
Error	−0.62%	−2.85%	+0.49%	+0.92%

Width (automatically)	3.84 m	12.54 m	8.29 m	9.84 m
Width (ground truth)	3.87 m	12.63 m	8.04 m	9.83 m
Error	−0.79%	−0.71%	+3.12%	+0.05%

**Table 6.** True positives, false positives and false negatives.

	**Case Study 1**	**Case Study 2**	**Case Study 3**	**Case Study 4**
Minimum width (cm)	70	70	70	70
Maximum width (cm)	100	100	100	120
Minimum height (cm)	200	200	200	200
Maximum height (cm)	240	240	240	240
Door candidates	23	57	106	54
Local percentile	95	85	85	95
Global percentile	85	80	75	85
Doors	3	11	9	1
True positives	3	9	7	1
False positives	0	0	7	2
False negatives	0	2	2	0

**Table 7.** Recall, precision and F1-Scores for the cases of study.

	**Case Study 1**	**Case Study 2**	**Case Study 3**	**Case Study 4**
Recall	1.00	0.82	0.78	1.00
Precision	1.00	1.00	0.50	0.33
F1-score	1.00	0.90	0.61	0.50
